# A case of mature cystic teratoma with intestinal structures harboring intestinal-type low-grade mucinous neoplasm

**DOI:** 10.1007/s13691-018-0321-6

**Published:** 2018-03-23

**Authors:** Maki Takao, Yasunori Yoshino, Ayumi Taguchi, Masaya Uno, Satoshi Okada, Nao Kino, Nobuaki Funata, Toshiharu Yasugi

**Affiliations:** 1 Gynecology, Tokyo Metropolitan Cancer and Infectious Diseases Center Komagome Hospital, 3-18-22 Honkomagome, Bunkyo-ku, Tokyo, 113-8677 Japan; 2 Pathology, Tokyo Metropolitan Cancer and Infectious Diseases Center Komagome Hospital, 3-18-22 Honkomagome, Bunkyo-ku, Tokyo, 113-8677 Japan

**Keywords:** Intestinal structures, Intestinal type, Mature cystic teratoma

## Abstract

The formation of gastrointestinal-type epithelium is found in 7–13% of mature cystic teratomas, which are the most common germ cell tumors of the ovary. Few cases harboring organized gastrointestinal tract formation have been reported, and a mucinous neoplasm arising in them is further rare. Here, we report a case of an ovarian mature cystic teratoma with intestinal structures harboring intestinal-type mucinous neoplasm, mimicking low-grade appendiceal mucinous cystadenoma. A 66-year-old female, with remarkably increased serum carcinoembryonic antigen (CEA) level, underwent total abdominal hysterectomy and bilateral salpingo-oophorectomy due to the ovarian tumor. The immunoprofile of the tumor showed CK7−/CK20+. We review the past literatures, and then consider that the existence of mucinous neoplasm should be kept in mind if we find elevated level of serum CEA and the organized gastrointestinal development in an ovary. The immunoprofile of CK7/CK20 is useful to determine the origin of mucinous tumors associated with mature cystic teratomas.

## Introduction

Mature cystic teratomas are the most common germ cell tumors of the ovary [1–3]. Histologically, they include elements of ectodermal origin mostly, endodermal origin in 32–72%, and gastrointestinal-type epithelium is found in 7–13% of cases [4–6]. However, few cases of organized gastrointestinal tract formation have been reported. Furthermore, a mucinous cystadenoma as secondary neoplasm arising from organized gastrointestinal tract in ovarian mature cystic teratoma is extremely rare [7].

Therefore, we report a very rare case of mature cystic teratoma with intestinal structures harboring intestinal-type mucinous neoplasm, with literature review.

## Case report

**Fig. 1:**
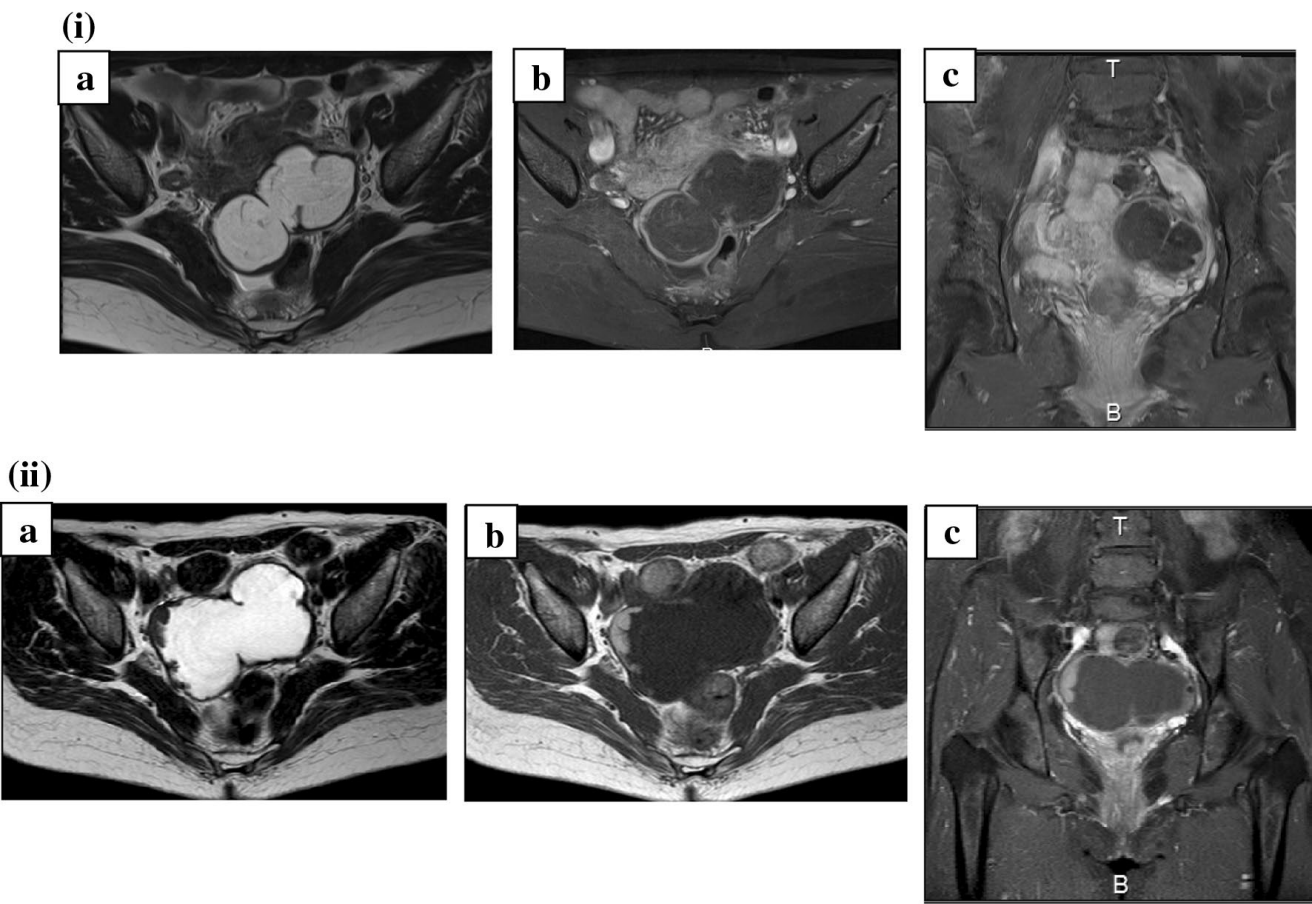
MRI findings. **i** At the first time. MRI showed a bilocular 8 cm mass with thick wall posterior to the uterus and demonstrated bleeding or mucinous component. *a* T2WI, *b* T1WI fat suppression, *c* Gd enhancement. **ii** 6 years later. MRI showed a 9×6 cm pelvic mass, including partial papillary nodules with contrast enhancement and little ascites. **a** T2WI, **b** T1WI, **c** Gd enhancement

A 66-year-old female, gravida 1, para 1, presented to our gynecologic unit with complaints of right lower abdominal pain. Magnetic resonance imaging (MRI) showed a bilocular 8 cm tumor with thick wall posterior to the uterus and demonstrated bleeding or mucinous component (Fig. 1i). A serum CEA level increased to 8.8 ng/ml (normal range 0.0–5.0 ng/ml). 2 months later, a size of the tumor was the same, and a serum CEA level was 8.4 ng/ml. We scheduled the gynecologic examination 1 year later, because she strongly hoped long interval of follow-up, but she did not show up and lost to follow-up.

6 years later, she presented to our hospital again, because elevated level of CEA (19.0 ng/ml) was found at other hospital. Gastroscopy and colonoscopy, which were performed at the previous hospital, revealed no abnormal findings. Her past medical history was unremarkable. Bimanual pelvic examination demonstrated a normal-sized uterus and cervix as well as a fist-sized cystic mass at Douglas cavum. The cervical cytology was negative. Transvaginal ultrasound identifies 8.5 × 6.0 × 5.0 cm cystic tumor with partial thick wall posterior to the uterus. MRI showed a 9 × 6 cm pelvic tumor, including partial papillary nodules with contrast enhancement and little amount of ascites (Fig. 1ii). The initial level of the serum CEA was 34.9 ng/ml.

A clinical impression of ovarian neoplasm, with suspicion of mucinous tumor of low malignant potential, was made and the patient underwent total abdominal hysterectomy and bilateral salpingo-oophorectomy. We recognized goose egg-sized left adnexal tumor, normal-sized uterus and right adnexae, the normal vermiform appendix, and no ascites during the surgery. Postoperative recovery was uncomplicated. The cytology of ascites was negative. The level of serum CEA decreased to normal (4.0 ng/ml) after the surgery. The patient received no further treatment and remains free of disease for 1.5 years.

**Fig. 2:**
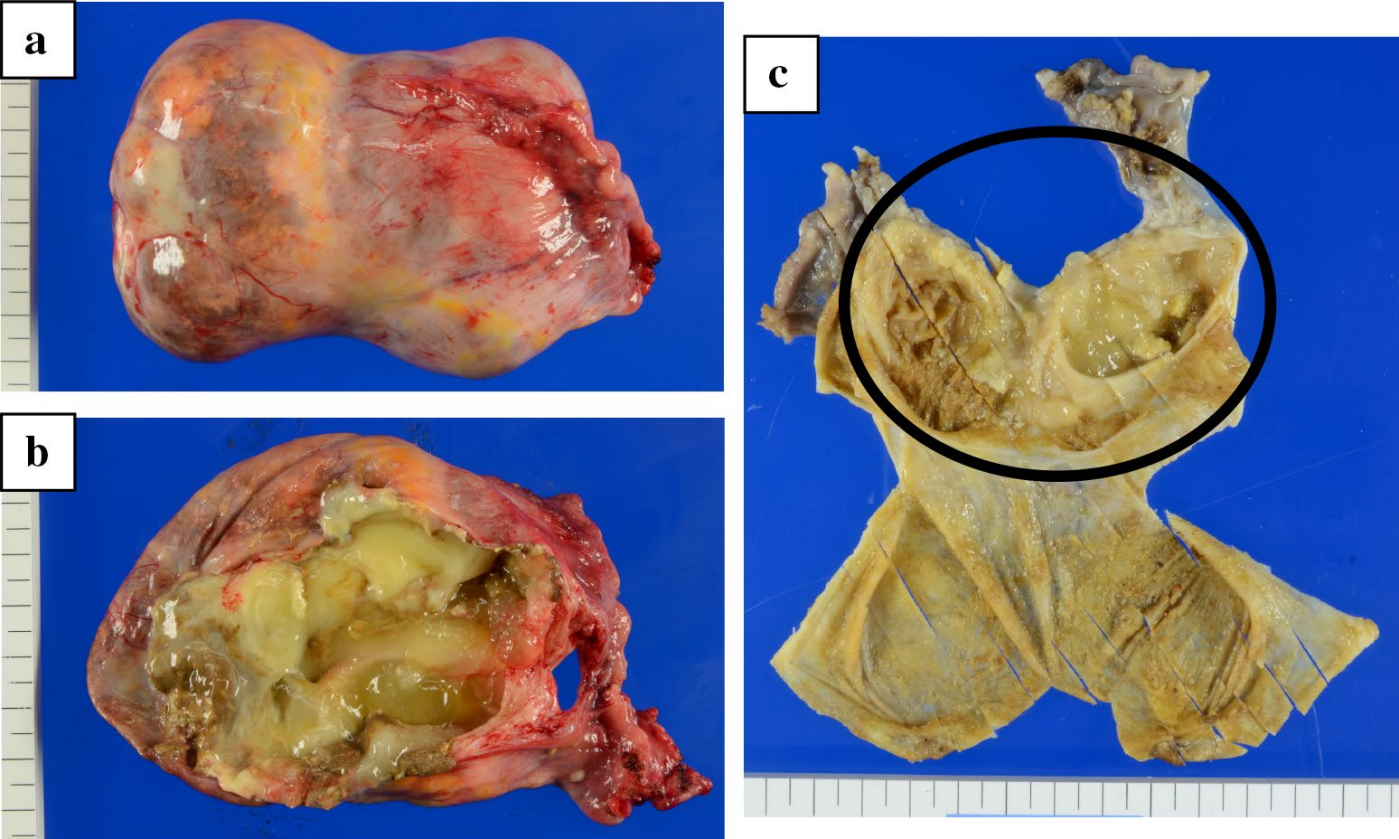
Gross appearance of the left ovary. **a** External surface of the left tumor was smooth, and not disrupted. **b, c** Cut section revealed partial thick wall, yellow gelatinous component, and a few hairs within the tumor, but a solid part did not exist

We described the macroscopic findings below. The surgical specimen consisted of a 9.5 × 6.5 × 4.0 cm—sized left ovarian tumor, normal uterus, and right adnexae. The external surface of the tumor was smooth, and not disrupted. The cut section revealed partial thick wall, yellow gelatinous component and a few hairs within the tumor, but a solid part did not exist (Fig. 2).

**Fig. 3:**
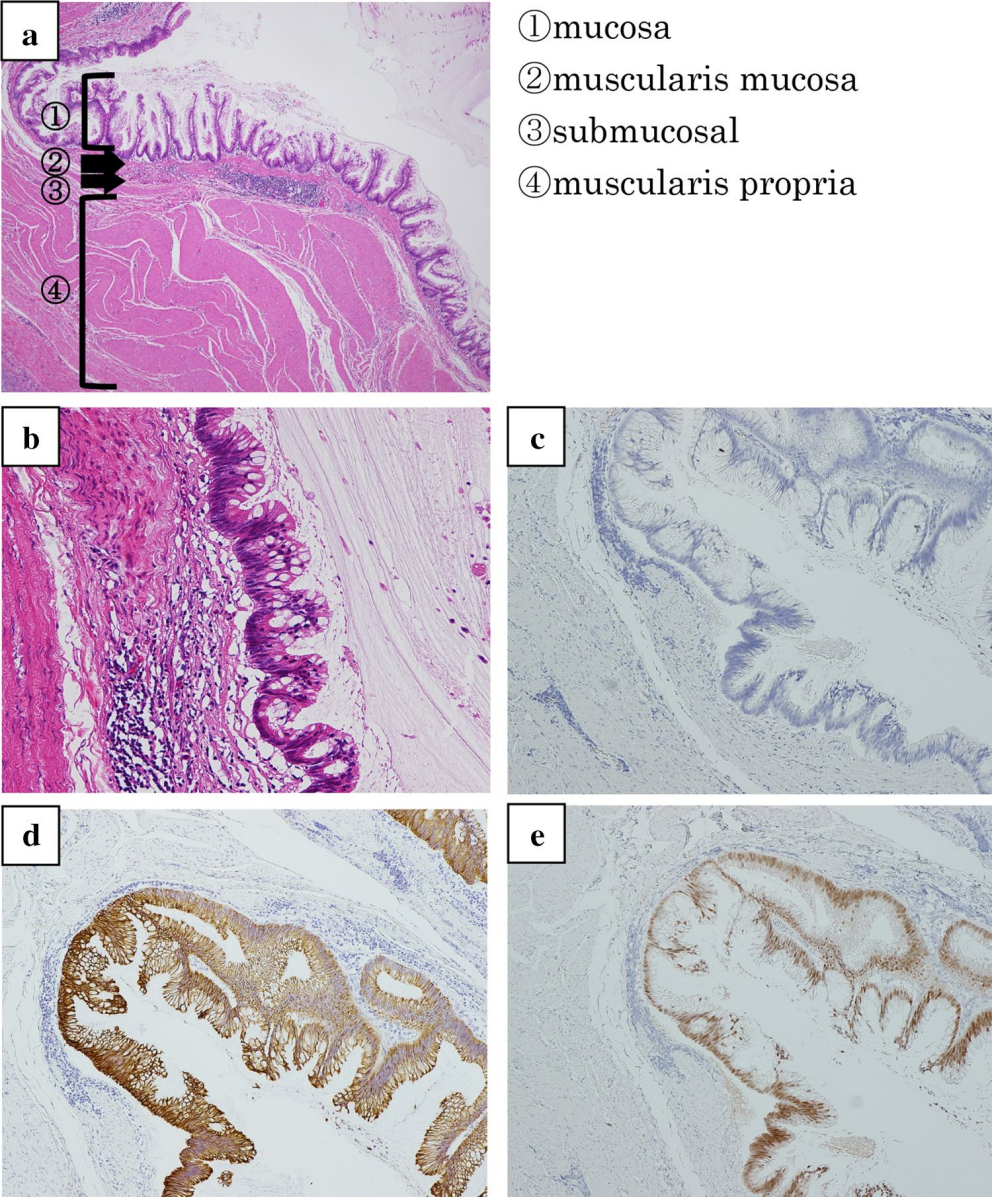
Microscopic appearance of the tumor (in the circular marks of Fig. 2c). **a, b** Wall consisted of four-layer structure, which was mucosa, muscularis mucosa, submucosal and muscularis propria. The cells of mucosa were negative for CK7 (**c**), positive for CK20 (**d**), and positive for CDX-2 (**e**)

On microscopic examination, sections from the cyst wall disclosed a mature cystic teratoma and contained mature tissue of stratified squamous epithelium, mucinous gland, and adipose tissue. The wall (in the circular marks of Fig. 2c) consisted of four-layer structure, which was mucosa, muscularis mucosa, submucosal, and muscularis propria. The mucosal cells were positive for PAS-Alcian blue stains, and the cells of muscularis mucosa and muscularis propria were positive for desmin stains. The cells showed highly dense, pseudostratified and disturbed nucleus, but mitosis was limited and there were no atypia and infiltration. They were positive for cytokeratin (CK) 20 and CDX (caudal-type homeobox transcription factor)-2, and negative for CK7 and p53 by immunohistochemistry stains, which corresponded with the features of intestinal epithelium (Fig. 3). The histopathological diagnosis was a mature cystic teratoma with intestinal structures harboring intestinal-type low-grade mucinous neoplasm of the left ovary, mimicking mucinous cystadenoma of appendix.

## Discussion

Mature cystic teratomas are the most common benign germ cell tumors of the ovary and usually consist of well-differentiated derivatives of three germ cell layers [1–3]. They include elements of ectodermal origin in 99–100%, mesodermal origin in 73–93%, and endodermal origin in 32–72% [4–6]. Gastrointestinal-type epithelium was found in 7–13% of cases, but organized gastrointestinal tract formation was seldom found [7]. To our knowledge, only eight cases have been reported in the English and Japanese literature [7–14] (Table 1). Woodfield et al. reported almost complete development of the gastrointestinal tract in a benign cystic teratoma first, and reviewed cases of the organized gastrointestinal development. They said that small and large bowel were reported most often and other unusual cases include evidence of extra cystic colon, esophagus, cecum with appendix, and ileum with appendix [10]. In addition, the mucinous cystadenoma originated from the colonic epithelium of the mature cystic teratoma like this case is further rare. Only Fujiwara [7] and Tang [11] have reported, respectively, to date.

**Table 1:** Reviews of past eight reports and this case

Author	Patient age	Tumor size (cm)	Tumor markers	Operation procedure	Features	Prognosis
Andrews HR (1912) [8]	29	The size of a tangerine orange	–	Bilateral cystectomy	Closely resembling intestine, coiled on itself, attached to the wall of the cyst by a structure resembling mesentery	–
Bernstine et al. (1959) [9]	51	17 × 12.5	–	–	Containing extra cystic colon with mesentery and meconium	–
Woodfield at al (1985) [10]	32	9 × 6 × 5	–	Right cystectomy	With almost complete development of the gastrointestinal tract (from esophagus to colon)	–
Fujiwara et al. (1995) [7]	35	5 × 7	–	Laparoscopic RSO	With formation of complete segments of intestinal wall, containing a benign mucinous cystadenoma of the appendiceal type	–
Tang P, et al (2003) [11]	16	18 × 12 × 9	CA125:89 U/ml	LSO	Containing a complete colonic wall in continuity with an endocervical-type mucinous cystadenoma	No recurrent
Arima K, et al. (2006) [12]	16	10	CA19-9:141 U/ml	Left cystectomy	Torsion of a MCT including intestine	–
Takano et al. (2015) [13]	20	23	CA125:36.2 U/ml	Bilateral cystectomy	Containing complete structures of colon and fundic gland	–
			SCC:2.8 ng/ml			
Ki, et al. (2016) [14]	54	5 × 5.6	–	Laparoscopic LSO	Containing complete colonic structure of the large intestine	–
This case	66	9.5 × 6.5 × 4.0	CEA:34.9 ng/ml	TAH + BSO	With intestinal structures harboring intestinal-type mucinous neoplasm, mimicking low-grade appendiceal mucinous cystadenoma	Free of disease for 1.5 years

*MCT* mature cystic teratoma, ― not mentioned, *RSO* right salpingo-oophorectomy, *LSO* left salpingo-oophorectomy, *TAH + BSO* total abdominal hysterectomy and bilateral salpingo-oophorectomy

We reviewed the MRI findings and discovered that partial papillary nodules with contrast enhancement had clarified after 6 years in spite of the tumor size was almost the same and the component was not changed. The pathological findings revealed that the papillary nodules were actually equal to folds of organized gastrointestinal tract. We guessed that the change had occurred and the mucinous cystadenoma had been developed from the gastrointestinal-type epithelium during 6 years slowly, which may correlated with the serum level of CEA. Elevated levels of CEA have also been reported in patients with neoplastic appendiceal mucoceles [15], which are rare and found in approximately 0.3% of appendectomy specimens. Neoplastic appendiceal mucoceles are called low-grade appendiceal mucinous neoplasm recently. They are pathologically benign, but potentially malignant because of their nature as the precursor to disseminated pseudomyxoma peritonei. The previous two reports were not mentioned about CEA, so we need more cases to reveal the relations.

Fujiwara also reported the case of mature cystic teratomas with complete intestinal wall differentiation which contained an intestinal-type adenocarcinoma [7]. Makihara et al. reported that a serum CEA level of 5.0 ng/ml or greater would be preoperative factors to distinguish malignant transformation in ovarian mature cystic teratomas [16]. We have to pay attention not to overlook the malignant transformation if the serum CEA level is increased with an ovarian tumor.

Most primary ovarian mucinous tumors are of surface epithelial–stromal origin and exhibit diffuse expression of CK7 combined with variable expression of CK20. This immunoprofile distinguishes them from gastrointestinal tract tumors secondarily involving the ovaries because the latter most often exhibit diffuse expression of CK20 coupled with lack of or limited expression of CK7 [17]. A literature of morphologic and immunophenotypic features of mucinous ovarian tumors showed that not only ovarian mucinous tumors associated with mature cystic teratomas but also tumors associated pseudomyxoma ovarii, tumors of germ cell origin and secondary or metastatic gastrointestinal tract tumors in the ovaries exhibit a CK7−/CK20+ profile [17]. That is why gastroscopy and colonoscopy including appendix is necessary for the patient if her ovarian tumor shows a CK7−/CK20+ profile. Recently, SATB2 is an identified protein with restricted expression in the glandular cells lining the lower gastrointestinal tract, so described as a sensitive and specific marker of colorectal epithelium [18, 19]. Some reports revealed that SATB2 was a useful marker for diagnosis of primary vs metastatic mucinous intestinal-type neoplasms and was highly sensitive in detecting lower gastrointestinal tract metastasis [18, 19]. In this case, SATB2 was not available at our hospital, but we did not find any observation in endoscopy before the surgery, and pseudomyxoma ovarii during the surgery. Therefore, we diagnosed a mature cystic teratoma with intestinal structures harboring intestinal-type low-grade mucinous neoplasm.

The detailed histopathological diagnosis is needed if we find elevated level of serum CEA and the organized gastrointestinal development in an ovary, and the immunoprofile of CK7/CK20 is useful to distinguish intestinal-type secondary neoplasms associated with mature cystic teratomas from primary ovarian mucinous tumors.
